# Predictive performance of BI-RADS magnetic resonance imaging descriptors in the context of suspicious (category 4) findings*

**DOI:** 10.1590/0100-3984.2015.0021

**Published:** 2016

**Authors:** João Ricardo Maltez de Almeida, André Boechat Gomes, Thomas Pitangueiras Barros, Paulo Eduardo Fahel, Mário de Seixas Rocha

**Affiliations:** 1 PhD, Physician, Department of Diagnostic Imaging, Clínica de Assistência à Mulher (CAM), Salvador, BA, Brazil.; 2 Physician, Department of Diagnostic Imaging, Clínica de Assistência à Mulher (CAM), Salvador, BA, Brazil.; 3 Professor, Biomedical Scientist, Escola Bahiana de Medicina e Saúde Pública, Salvador, BA, Brazil.; 4 Physician, Department of Pathology, Clínica de Assistência à Mulher (CAM), Salvador, BA, Brazil.; 5 PhD, Assistant Professor of Medicine, Escola Bahiana de Medicina e Saúde Pública, Salvador, BA, Brazil.

**Keywords:** Magnetic resonance imaging, Breast neoplasms, Predictive value of tests, Likelihood functions

## Abstract

**Objective:**

To determine the positive predictive value (PPV) and likelihood ratio for magnetic resonance imaging (MRI) characteristics of category 4 lesions, as described in the Breast Imaging Reporting and Data System (BI-RADS^®^) lexicon, as well as to test the predictive performance of the descriptors using multivariate analysis and the area under the curve derived from a receiver operating characteristic (ROC) curve.

**Materials and Methods:**

This was a double-blind review study of 121 suspicious findings from 98 women examined between 2009 and 2013. The terminology was based on the 2013 edition of the BI-RADS.

**Results:**

Of the 121 suspicious findings, 53 (43.8%) were proven to be malignant lesions, with no significant difference between mass and non-mass enhancement (*p* = 0.846). The PPVs were highest for masses with a spiculated margin (71%) and round shape (63%), whereas segmental distribution achieved a high PPV (80%) for non-mass enhancement. Kinetic analyses performed poorly, except for type 3 curves applied to masses (PPV of 73%). Logistic regression models were significant for both patterns, although the results were better for masses, particularly when kinetic assessments were included (*p* = 0.015; pseudo *R^2^* = 0.48; area under the curve = 90%).

**Conclusion:**

Some BI-RADS MRI descriptors have high PPV and good predictive performance-as demonstrated by ROC curve and multivariate analysis-when applied to BI-RADS category 4 findings. This may allow future stratification of this category.

## Abstract (pt)

**Objetivo:**

Determinar o valor preditivo positivo (VPP) e a razão de verossimilhança positiva de características de ressonância magnética (RM) de lesões da categoria 4, como descritas no léxico do *Breast Imaging Reporting and Data System* (BI-RADS^®^), e testar o desempenho preditivo dos descritores por meio de análise multivariada e área sob a curva derivada da curva receiver operating characteristic (ROC).

**Materiais e Métodos:**

Foi realizado um estudo revisional duplo-cego de 121 achados suspeitos em 98 mulheres examinadas entre 2009 e 2013. A terminologia foi baseada na edição de 2013 do BI-RADS.

**Resultados:**

Dos 121 achados suspeitos, 53 (43,8%) eram de fato lesões malignas, sem diferença significativa entre nódulos e realce não nodular (*p* = 0,846). Nódulos com margem espiculada (71%) e forma redonda (63%) apresentaram os maiores VPPs, ao passo que a distribuição segmentar teve alto VPP para realce não nodular (80%). Apenas a curva cinética do tipo 3 teve bom desempenho quando aplicada a nódulos (VPP = 73%). Modelos de regressão logística foram significantes para os dois padrões principais, embora os nódulos tenham apresentado resultados melhores, particularmente com a introdução da análise cinética (*p* = 0,015; pseudo-*R^2^* = 0,48; área sob a curva = 90%).

**Conclusão:**

Alguns descritores de RM do BI-RADS têm alto VPP e bom desempenho preditivo - demonstrado por curva ROC e análise multivariada - quando aplicados a achados da categoria 4 segundo o BI-RADS. Isso pode permitir futura estratificação dessa categoria.

## INTRODUCTION

Mainly because of its high sensitivity, magnetic resonance imaging (MRI) has progressively attained a prominent position in the diagnosis of breast cancer and screening of high-risk women^(1,2)^. That triggered the widespread dissemination of the method and brought challenges to referring physicians, particularly breast care specialists and oncologists; old treatment paradigms had to be reassessed in light of the (relatively) new technique, leading to a fair amount of uncertainty^(3,4)^.

One frequent claim concerns the proportionately low specificity of breast MRI when compared with mammography and ultrasound^(5,6)^. This argument, albeit fallacious-given that the slightly lower specificity of breast MRI is partly credited to its unparalleled sensitivity-is frequently coupled with questions regarding the high number of false-positive results reported^(7,8)^. These potential limitations would increase the numbers of unnecessary operations and aggressive procedures applied to any suspicious abnormality^(3)^. As a consequence, the capability of MRI to distinguish between benign and malignant lesions with accuracy has always been under scrutiny within the medical community^(9)^.

In an effort to address some of these matters, the American College of Radiology (ACR) Breast Imaging Reporting and Data System (BI-RADS^®^) included MRI in its two latest editions^(10,11)^. The concepts of standardized terminology (lexicon) and general assessment categories were adjusted to the particularities of MRI, similar to what had previously been done for mammography and ultrasonography. Nevertheless, stratification guidelines for MRI category 4 findings-which have estimated cancer likelihoods ranging from > 2% to < 95%^(11)^-were not issued, in contrast to what is already the norm for the other imaging methods^(12,13)^. In order to achieve this feat, it is paramount to examine the predictive values of individual descriptors in this particular context.

The aim of this study is to establish the positive predictive values (PPVs) and positive likelihood ratios (PLRs) for BI-RADS descriptors applied to category 4 abnormalities. We also identified the most cancer-related features and probed them in a multivariate model.

## MATERIALS AND METHODS

This retrospective cross-sectional study stems from a graduate (*sensu stricto*) project sponsored by an academic medical institution and a regional private referral clinic in women's healthcare. The independent review board of the medical school approved the study (Report no. 518.466) and waived the requirement for written informed consent.

### Study design and population

Between November 2009 and December 2013, 1973 breast MRI studies were performed at our private practice. Of those 1973 studies, 238 (12.06%) revealed one or more suspicious findings (BI-RADS 4 lesions). It is our standard practice to schedule visits with premenopausal women between the 5th and 14th days of their menstrual cycle. This protocol is bypassed only when the requesting physician considers the situation urgent.

**Figure 1:**
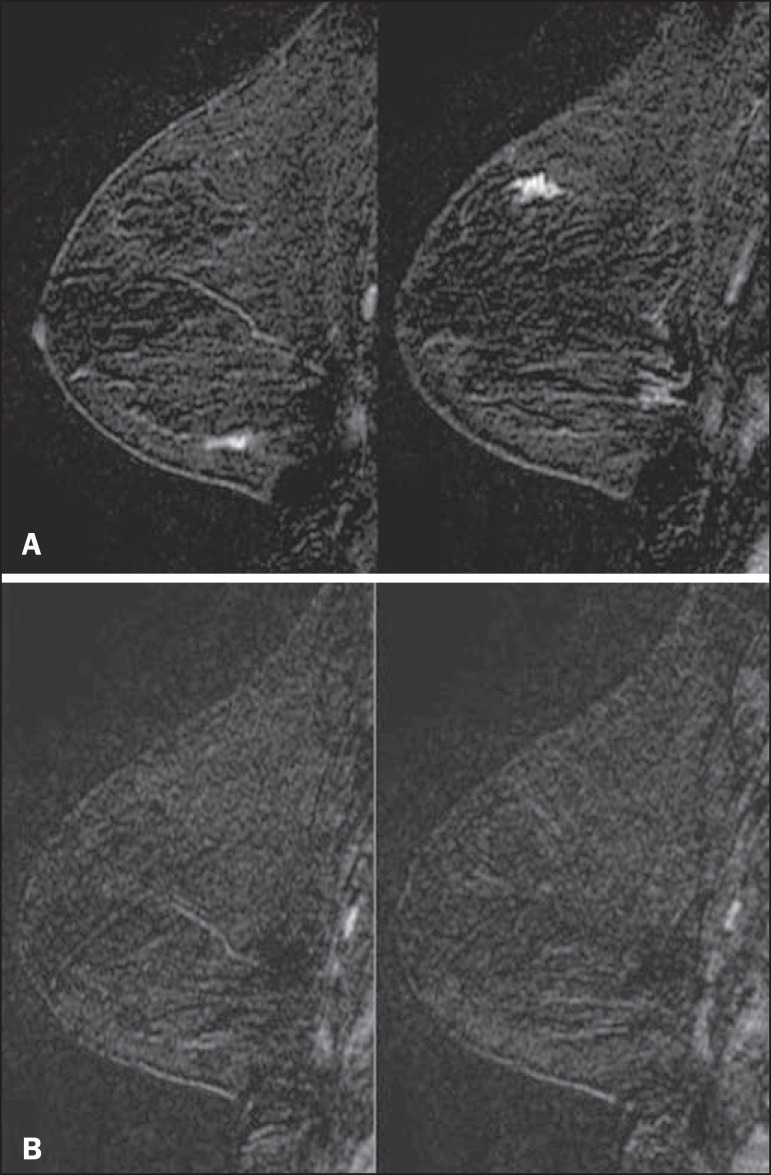
Patient presenting with synchronous areas of non-mass enhancement on sagittal T1-weighted post-contrast fat-saturated images after subtraction. Despite being considered suspicious, the findings were followed only by MRI. **A:** First examination, showing linear and focal areas of enhancement on the right breast, at approximately 6 and 12 o'clock, respectively, without representation on non-contrasted images (not shown). **B:** Control scan obtained six months after the first examination, showing no areas of enhancement. After 2 years of followup, the patient displayed no new abnormalities and the areas were categorized as probable functional asymmetric enhancement.

Our information technology team restored and anonymized 158 MRI studies to an independent image databank linked to a restricted version of the electronic medical record database of the institution (80 records could not be accessed due to random data corruption secondary to defective media). Exclusion criteria were: Records related to repeated investigations of the same patient without new findings were excluded (only the first one was included in the analyses), as were those related to small lesions (less than 5 mm), those including reports of pronounced image artifacts (such as patient movement and field inhomogeneity), and those in which there was no histopathologic outcome or adequate site correspondence between the MRI finding(s) and the pathologic description. We decided to include 13 records related to lesions with diagnostic cytology only, because they were conclusive and had been monitored for at least two years. We also included one record related to a patient with two suspicious areas of enhancement that disappeared during follow-up and were not more aggressively investigated (Figure 1). We excluded a total of 60 records-42 due to lack of follow-up, 16 because of image artifacts, and two because the lesions were considered foci. Therefore, the final study sample comprised 98 patients, with ages ranging from 28 to 88 years (mean of 51.5 years), among whom 121 findings were classified as BI-RADS 4 lesions.

### MRI protocol

Images were acquired in a single 1.5 T MRI scanner (Signa Excite HdxT; GE Healthcare, Madison, WI, USA) with a dedicated, bilateral, 8-channel phased-array coil, while the patient was in the prone position. The standard protocol at the facility consists of sagittal T1-weighted fast spin-echo sequences (repetition time/echo time [TR/TE], 400/15; echo-train length, 5; bandwidth, 41.7 MHz; number of excitations, 1; matrix size, 320 × 224; field of view, 200 × 200 mm; slice thickness, 4 mm; intersection gap, 0.5), sagittal fat-suppressed T2-weighted images (TR/TE, 4500/85; echo-train length, 17; bandwidth, 25.0 MHz; number of excitations, 3; matrix size, 256 × 192; field of view, 200 × 200 mm; slice thickness, 4 mm; intersection gap, 0.5), and dynamic contrast-enhanced MRI acquisitions with three-dimensional fast spoiled gradient-recalled echo sequence using Volume Imaging for BReast Assessment (VIBRANT) parallel imaging (GE Healthcare) in the sagittal plane (TR/TE, 5.5/2.7; flip angle, 15º; bandwidth, 50.0; number of excitations, 1; matrix size, 320 × 192; field of view, 200 × 200 mm; slice thickness, 3 mm; intersection gap, 0 mm; reduction factor, 2). The dynamic study is composed of pre-contrast images followed by five acquisitions spaced at 75-s intervals after bolus injection of 0.1 mmol/kg of body weight of gadoterate meglumine (Dotarem; Guerbet, Paris, France). We also acquire a late axial isotropic sequence: single-phase VIBRANT (TR/TE, 5.0/2.4; flip angle, 15º; bandwidth, 62.5; number of excitations, 1; matrix size, 350 × 350; field of view, 340 × 340 mm; slice thickness, 1 mm; intersection gap, 0 mm; reduction factor, 2).

### Image analysis and data collection

The selected examinations were stored in an offline Advantage Windows workstation, version 4.4, with the Functool Software Package (GE Healthcare) for post-processing. Two examiners with at least 1000 breast MRI readings to their credit, blinded to the clinical data and pathologic outcomes, independently reviewed the images and described suspicious findings strictly according to the BI-RADS MRI lexicon. No studies were reclassified under a different BI-RADS category after review. Subsequently, the reviewers reanalyzed divergent descriptions and achieved a consensus in all cases. The same procedure was implemented for the semi-quantitative kinetic assessment, with regions of interest (containing at least four pixels) positioned over the most suspicious area of enhancement, and the kinetic curve generated was classified as type 1, 2 or 3, corresponding to the delayed phases (persistent, plateau, and washout, respectively).

Mass and non-mass enhancement (NME) were equally described in terms of the internal enhancement pattern, T2 signal intensity, and type of curve, mostly based on the terminology of the 2013 (5th) edition of the ACR BI-RADS^(11)^. Masses were termed specifically for shape and margin, while symmetry (when applicable) and distribution were defined for NME, because we opted to keep symmetry as an NME finding, according to the 2003 edition of the ACR BI-RADS^(10)^.

### Pathologic outcome

A pathologist specialized in breast diseases, with more than 10 years of experience, reevaluated and dichotomized the available reports as positive or negative for malignancy. By doing so, lesions considered to be indeterminate or high-risk in nature (atypical findings, lobular neoplasia, complex sclerosis, or papillary lesions) were categorized as non-malignant. In cases with mixed histological features, the most aggressive pattern was used as the grouping indicator. The 13 cases investigated only by cytology were also validated, because they were compatible with imaging findings and showed benign evolution during follow-up.

### Statistical analysis

Lexicon descriptors were categorized and correlated with the outcome (malignant or non-malignant). We considered positive cancer diagnoses as true positives for individual descriptors and, based on that assumption, PPVs and PLRs were calculated with 95% confidence intervals (95% CIs).

The association between independent descriptors and cancer frequencies were explored using the chi-square test of independence and, when fewer than five occurrences were expected, by Fisher's exact test. We adopted a 5% level of significance (*p* < 0.05) for two-tailed tests. Predictor variables were assessed by univariate logistic regression, and those with a *p* < 0.20 were inserted into separate multivariate predictive models for masses and NME. Each covariate had a minimum of six least frequent outcomes, and, because there was more than one finding for some participants (average of 1.24 findings), the intracluster correlation coefficient was employed. In addition, should any significant predictor demonstrate relevant collinearity-defined as variance inflation factor above 3.5-it would be discarded. Odds ratios (ORs) and 95% CIs were calculated for significant predictors. The fit and explained variability of the models were assessed by the Hosmer-Lemeshow test and pseudo *R^2^*. We also generated receiver operating characteristic (ROC) curve for the best model and derived its area under the curve (AUC). For those computations, we used Stata statistical software, version 12.0 (StataCorp LP; College Station, TX, USA).

## RESULTS

### Patients and subjects

We evaluated 121 suspicious MRI findings among 98 patients. Of those 121 findings, 53 (43.8%) were determined to be malignant lesions, including 12 invasive carcinomas not otherwise specified, 13 invasive ductal carcinomas, 6 invasive lobular carcinomas, 2 neuroendocrine tumors, 2 mucinous carcinomas, 2 invasive tubular lesions, and 16 ductal carcinomas in situ. The remaining 68 (56.2%) were determined to be non-malignant lesions, of which 17 (25.0%) were classified as indeterminate/high risk lesions-comprising 1 case of atypical columnar hyperplasia; 5 cases of unspecified atypical findings; 1 complex sclerosing lesion; and 10 papillary lesions-whereas 51 (75.0%) were classified as benign-including 15 fibroadenomas; 17 proliferative or nonproliferative fibrocystic changes; 11 inflammatory conditions; 2 pseudoangiomatous stromal hyperplasias; 1 pronounced peritumoral angiogenesis; 1 fibrous nodule; 2 conclusive cases of negative cytologic studies; and 2 enhancement abnormalities that progressively disappeared.

The pathologic material originated from 21 (17.4%) mammography or ultrasound-guided core biopsies, 3 (2.5%) mammography-guided mammotome excisions, 1 (0.8%) MRI-guided mammotome excision, 12 (9.9%) fine-needleaspirations, and 1 (0.8%) nipple discharge study (the last two with additional 2-year imaging follow-up). Of the 121 suspicious MRI findings, the majority-81 (66.9%)-were defined on the basis of samples obtained during surgical procedures (excisional biopsy or treatment). No pathologic confirmation was obtained for 2 (1.7%) of the suspicious MRI findings: both were NMEs observed in a single participant and progressively disappeared during MRI follow-up (therefore being considered benign functional abnormalities). We did not find a significant difference between surgical and nonsurgical procedures in terms of the cancer yield (*p* = 0.084 from Fisher's exact test).

### Cancer likelihood based on morphology and T2 signal intensity

The main enhancement patterns demonstrated similar frequencies of malignancy, 24 (42.9%) of the 56 masses and 29 (44.6%) of the 65 NMEs being categorized as positive (*p* = 0.846 from the chi-square test), as shown in Tables 1 and 2. However, 14 (87.5%) of the 16 ductal carcinomas in situ presented as NMEs, whereas the majority-22 (59.5%)-of the 37 invasive cancers appeared as masses.

**Table 1:** Characteristics of BI-RADS 4 findings classified as masses.

	Lesions*		Positive lesions		
Descriptor	*N (%)*		*N*	PPV^†^ [95% CI]	PLR^†^ [95% CI]
Mass	56 (46.28)		24	43 [30–57]	0.96 [0.65–1.42]
Shape					
Oval	19 (33.93)		4	21 [6–46]	0.36 [0.14–0.94]
Round	16 (28.57)		10	63 [35–85]	2.22 [0.94–5.27]
Irregular	21 (37.50)		10	48 [26–70]	1.21 [0.62–2.38]
Margin					
Circumscribed	7 (12.50)		1	14 [0.4–58]	0.22 [0.03–1.73]
Irregular	42 (75.00)		18	43 [28–59]	1.00 [0.74–1.36]
Spiculated	7 (12.50)		5	71 [29–96]	3.33 [0.71–15.7]
Internal enhancement					
Homogeneous	26 (46.43)		7	27 [12–48]	0.49 [0.25–0.98]
Heterogeneous	17 (30.36)		10	59 [33–82]	1.90 [0.85–4.27]
Rim enhancement	13 (23.21)		7	54 [5–81]	1.56 [0.60–4.04]
Dark internal septations	0		0	—	—
T2 signal					
Low	18 (32.14)		10	56 [31–79]	1.67 [0.78–3.58]
High	38 (67.86)		14	37 [22–54]	0.78 [0.53–1.15]

* Data in parentheses are percentages of the total number of masses (*n* = 56), except on the first row, where they are percentages of the total number of lesions (*n* = 121);

† Expressed as %; —, Values that could not be calculated due to a lack of the finding in the study sample.

**Table 2:** Characteristics of BI-RADS 4 lesions classified as non-mass enhancement.

	Lesions*		Positive lesions		
Descriptor	*N (%)*		*N*	PPV^†^ [95% CI]	PLR^†^ [95% CI]
Non-mass enhancement	65 (53.72)		29	45 [32–58]	1.03 [0.74–1.44]
Distribution and symmetry					
Focal	25 (38.46)		7	28 [12–49]	0.48 [0.23–1.00]
Linear	11 (16.92)		4	36 [11–69]	0.71 [0.23–2.19]
Segmental	15 (23.08)		12	80 [52–96]	4.97 [1.55–15.90]
Regional	4 (6.15)		1	25 [63–81]	0.41 [0.05–3.77]
Multiple regions	1 (1.54)		1	100 [0.03–100]	—
Diffuse	0		0	—	—
Symmetric	0		0	—	—
Asymmetric	9 (13.85)		4	44 [14–79]	0.99 [0.29–3.37]
Internal enhancement					
Homogeneous	8 (12.31)		1	13 [32–53]	0.18 [0.02–1.36]
Heterogeneous	54 (83.08)		25	46 [33–60]	1.07 [0.86–1.33]
Clumped	3 (4.62)		3	100 [29–100]	—
T2 signal					
Low	47 (72.31)		21	45 [30–60]	1.00 [0.74–1.36]
High	18 (27.69)		8	44 [22–69]	0.99 [0.45–2.19]

* Data in parentheses are percentages of the total number of non-mass enhancement (*n* = 65), except on the first row, where they are percentages of the total number of lesions (*n* = 121).

† Expressed as %. —, Values that could not be calculated due to a lack of the finding in the study sample.

The individual mass descriptors with the highest PPVs were spiculated margin (71%) and round shape (63%), both of which had equally high PLRs (Table 1). In the NME group, segmental distribution had a high PPV (80%), there was one case of multiple regions of enhancement, and there were three cases of clumped internal pattern, all four cases being categorized as positive for malignancy (Table 2).

In the univariate analyses of descriptors for masses, T2 signal intensity did not achieve the cutoff significance level to be introduced into the multivariate logistic regression (*p* = 0.252). The model including shape, margin, and internal enhancement as predictor variables could significantly distinguish between malignant and non-malignant lesions (*p* = 0.038; pseudo *R^2^* = 0.24), round shape independently reaching significance (OR: 12.91; 95% CI: 2.33-71.45; *p* = 0.003) and rim enhancement reaching marginal significance (OR: 7.15; 95% CI: 0.96-53.19; *p* = 0.055).

NME-related terms performed more poorly, only distribution and internal enhancement being eligible for multivariate modeling. The logistic regression analysis was significant (*p* = 0.031; pseudo *R^2^* = 0.16) mainly because multiple regions of enhancement and clumped internal pattern were seen in a small number of all positive cases.

### Cancer likelihood based on enhancement kinetics

Semi-quantitative kinetic analyses displayed different levels of performance when considered in association with the main patterns of enhancement. Washout (type 3) curves did have a high PPV (73%) but only when associated with masses; as for NME, none of the kinetic curves were substantially linked to malignancy (Table 3).

**Table 3:** Enhancement characteristics of BI-RADS 4 lesions, based on semi-quantitative kinetic analysis.

	Lesions*		Positive lesions		
Type of curve	*N (%)*		*N*	PPV^†^ [95% CI]	PLR^†^ [95% CI]
Mass (*n* = 51)					
Type 1	8 (15.69)		3	38 [0.09–76]	0.73 [0.20–2.74]
Type 2	28 (54.90)		9	32 [16–52]	0.58 [0.33–1.02]
Type 3	15 (29.41)		11	73 [45–92]	3.35 [1.23–9.12]
Non-mass enhancement (*n* = 62)					
Type 1	27 (43.55)		12	44 [26–65]	0.91 [0.51–1.61]
Type 2	29 (46.77)		14	48 [29–68]	1.06 [0.63–1.81]
Type 3	6 (9.68)		3	50 [11.8–88.2]	1.14 [0.25–5.21]

* Data in parentheses are percentages of the total number of lesions by main enhancement pattern: mass and non-mass enhancement.

† Expressed as %.

**Figure 2:**
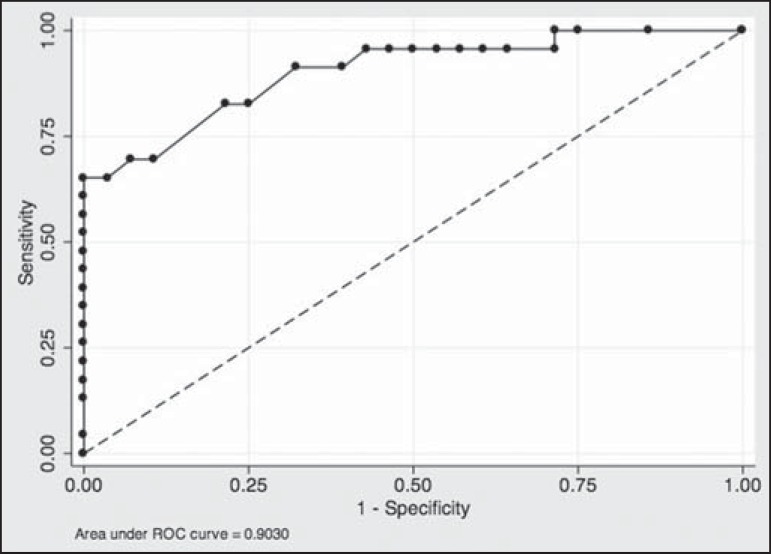
Receiver operating characteristic curve generated from the logistic regression model for nodules. Shape, margin, internal enhancement, and kinetic curve assessment were the independent variables. The performance of the model was considered highly satisfactory because it achieved an area under the curve of 90%.

When the kinetic analyses were inserted into the predictive models, the one for NME showed no significant improvement. Masses, however, displayed better model fit, with increased significance and explained variability (*p* = 0.015; pseudo *R^2^* = 0.48), with four significant adjusted descriptors: round shape, type 3 curve, heterogeneous enhancement, and rim enhancement (Table 4). The ROC curve for this model showed an AUC of 90% (Figure 2).

**Table 4:** Multivariate model for mass descriptors applied to BI-RADS 4 lesions with kinetic curve assessment.

	Beta		
Predictor variable	coefficient	Odds ratio [95% CI]	*P*
Shape			
Oval	0.00	1.00 (reference)	0.051
Round	4.35	77.66 [2.21–2,732.03]	0.017
Irregular	0.69	1.99 [0.30–13.03]	0.474
Margin			
Circumscribed	0.00	1.00 (reference)	0.363
Irregular	3.00	20.14 [0.18–2,271.07]	0.21
Spiculated	3.89	48.98 [0.23–10,434.72]	0.16
Internal enhancement			
Homogeneous	0.00	1.00 (reference)	0.039
Heterogeneous	3.33	27.87 [2.10–370.17]	0.012
Rim enhancement	3.54	34.39 [1.29–918.38]	0.035
Kinetic curve			
Type 1	0.00	1.00 (reference)	< 0.001
Type 2	–1.74	0.18 [0.01–2.67]	0.211
Type 3	2.25	9.47 [1.27–70.35]	0.028

## DISCUSSION

The capability of MRI to differentiate between malignant and non-malignant findings has occasionally been called into question because of the overlapping characteristics between the two and the variable predictive values^(14-16)^. Suspicious (category 4) lesions are even more problematic, because they present, by definition, an unacceptably wide range of malignancy likelihoods^(11,17)^. In this study, we displayed positive predictive measures for each BI-RADS descriptor and applied simple multivariate logistic regression models to test their performance in this context. The observed results indicate that stratification of category 4 findings based on lexicon-established terminology might also possible for MRI.

There are a large number of works about predictive values of breast MRI characteristics^(15,18,19)^. However, to our knowledge, this is the first study to focus exclusively on BIRADS 4 lesions. Most studies of this topic have investigated categories 4 and 5 together, and some have also included categories 0 through 3^(14,15)^. The reader should bear this in mind if comparing our results with those of other authors.

The prevalence of malignancy in our sample (43.8%) is within the range specified by the BI-RADS as one of the MRI screening benchmarks^(11)^. The BI-RADS stipulates that the biopsy yield of malignancy (PPV_3_)-comprising categories 4 and 5-should be within the 20-50% range. Our finding is also consistent with most of the reported values, which range from 20% to 60%^(20,21)^. Nevertheless, to account for pretest probability, we provided the corresponding likelihood ratios.

We detected almost equivalent frequencies of malignancy between masses and NMEs, similarly to Liberman et al.^(18)^. However, the vast majority of intraductal carcinomas (87.5%) appeared as NME, in accordance with most of the published results, in which reported frequencies range from 53.8% to 90%^(18,22,23)^. Nevertheless, among all of the non-mass findings, ductal carcinoma *in situ* was in the minority: only 14 out of 29 (48.3%) had no invasive component. One plausible explanation for this is that when more than one pathologic feature was found in a single lesion, we focused only on the most aggressive feature.

The single mass-specific descriptor with the highest predictive measures was spiculated margin (PPV of 71%), because this term is often considered highly suggestive of malignancy. Unexpectedly, round shape was the second most predictive descriptor (PPV of 63%). Round shape is variably associated with cancers, particularly of the triple-negative subtype, depending on the imaging method studied. Liberman et al.^(24)^ noted a 42% frequency of malignancy as a function of that descriptor applied to mammography. In contrast, Rahbar et al.^(25)^ found that only 6% of the round masses identified by ultrasonography were malignant. The other pattern-specific terms in our sample, including irregular margin, had poorer predictive performances, with PPVs below 60%.

**Figure 3:**
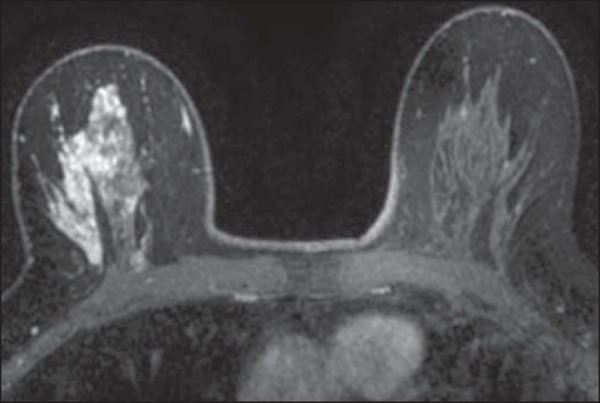
Multiple regions of enhancement in the right breast on an axial T1-weighted fat-saturated post-contrast image. The lesion was biopsied, and the patient subsequently underwent surgery, which confirmed the diagnosis of multicentric invasive lobular carcinoma.

There were two non-mass descriptors with PPVs of 100%-clumped internal pattern and multiple regions of enhancement-the latter representing an invasive multicentric lobular carcinoma (Figure 3). Although the first term is commonly linked to higher cancer frequencies^(14,26)^, the small number of both findings in our sample precludes further conclusions. Segmental distribution also had substantial predictive power (PPV of 80%), although not enough to be included as a category 5 descriptor, in contrast with the findings of Kuhl et al.^(27)^.

Logistic regression analyses showed good performance for masses, especially when kinetic curves were considered, as demonstrated by the ROC curve (AUC of 90%), although not for NME. These findings are somewhat similar to those reported by Yamaguchi et al.^(28)^, who, like us, found that only a few of the adjusted variables were significantly predictive. To account for correlated BI-RADS descriptors, as shown by Benndorf et al.^(29)^, we ran model diagnostics and excluded predictors with high degrees of collinearity.

Our group elected to define the kinetic signal intensity graphic only by the composite description of the early and delayed enhancement phases, characterized as type 1, 2, or 3 curves. By taking this approach, analogous to that proposed by Kuhl et al.^(30)^, we avoided the independent appraisal of the two distinct phases, which departs from our clinical routine. We observed a likelihood of malignancy for type 3 curves higher than that previously reported^(15,30)^, although only when associated with masses.

This study has certain limitations. We excluded a considerable number of examinations, for various causes. However, most were excluded because of digital storage issues. Although we do not identify any systematic bias, because the data corruption was apparently random, we acknowledge that there is a potential for unknown bias. For some of the descriptors, the data were insufficient to generate reliable PPVs and PLRs with manageable 95% CIs. In addition, the PPVs presented here are valid only when considering the stratification of BI-RADS 4 findings (4A, 4B, and 4C) and should not be generalized to other categories. Furthermore, some terms-"dark internal septations", "symmetric", "diffuse", and "clustered ring"-were not utilized in this sample. We also chose to keep 15 findings (12.4%) that had no histopathologic validation. That was done in order to minimize selection bias and avoid an increase in the number of positive outcomes, which would have occurred had we kept only the findings submitted to aggressive investigation. The small number of MRI-guided procedures was an expected shortcoming, despite our facility being the only center in the region that is equipped for such procedures. We also had some correlated observations, as some patients presented more than one finding. However, we corrected them statistically by running multivariate models with cluster-robust standard errors.

## CONCLUSION

We have shown that PPVs of certain BI-RADS descriptors can be used to discriminate malignant outcomes in the particular context of MRI category 4 abnormalities. The results presented here suggest that stratification of these lesions into low (4A), moderate (4B), and high (4C) suspicion subgroups is feasible and might be achieved in future editions of the ACR BI-RADS as larger studies are published.
